# Effect of molecular architecture on ring polymer dynamics in semidilute linear polymer solutions

**DOI:** 10.1038/s41467-019-09627-7

**Published:** 2019-04-15

**Authors:** Yuecheng Zhou, Kai-Wen Hsiao, Kathryn E. Regan, Dejie Kong, Gregory B. McKenna, Rae M. Robertson-Anderson, Charles M. Schroeder

**Affiliations:** 1 Department of Materials Science and Engineering, University of Illinois at Urbana-Champaign, Urbana, IL 61801 USA; 2 Beckman Institute for Advanced Science and Technology, University of Illinois at Urbana-Champaign, Urbana, IL 61801 USA; 3 Department of Chemical and Biomolecular Engineering, University of Illinois at Urbana-Champaign, Urbana, IL 61801 USA; 4 Department of Physics, University of San Diego, San Diego, CA 92110 USA; 5 Department of Chemical Engineering, Texas Tech University, Lubbock, TX 79409 USA

## Abstract

Understanding the dynamics of ring polymers is a particularly challenging yet interesting problem in soft materials. Despite recent progress, a complete understanding of the nonequilibrium behavior of ring polymers has not yet been achieved. In this work, we directly observe the flow dynamics of DNA-based rings in semidilute linear polymer solutions using single molecule techniques. Our results reveal strikingly large conformational fluctuations of rings in extensional flow long after the initial transient stretching process has terminated, which is observed even at extremely low concentrations (0.025 c^*^) of linear polymers in the background solution. The magnitudes and characteristic timescales of ring conformational fluctuations are determined as functions of flow strength and polymer concentration. Our results suggest that ring conformational fluctuations arise due to transient threading of linear polymers through open ring chains stretching in flow.

## Abstract (web summary)

Understanding the dynamics of ring polymers is challenging are despite recent progress, understanding of the nonequilibrium behavior of ring polymers has not yet been achieved. Here, the authors directly observe the flow dynamics of DNA-based rings in semidilute linear polymer solutions using single molecule techniques.

## Introduction

Ring polymers are intriguing materials with unique properties due to a topologically enclosed macromolecular structure with no beginning or end^1^. With regards to biological molecules, it has been proposed that the topological constraints of ring (or circular) polymers can potentially inform chromatin folding and genome organization^2^. In the realm of synthetic polymers, ring polymer topology has been leveraged to create new sustainable materials. In recent work, synthetic ring polymers based on cyclic poly(phthalaldehyde) (cPPA) show high levels of purity and stability^3^. Interestingly, polymer degradation (depolymerization) of cPPA can be precisely triggered by heating or by applying an external chemical stimulus. In this way, cPPA serves as a promising candidate for next-generation plastics that disintegrate on demand^4^.

Achieving a clear understanding of ring polymer dynamics is important for fundamental polymer physics. Experimental efforts to study high purity ring polymer melts began many years ago. Early work focused on the properties of synthetic ring polymers based on polystyrene and polybutadiene using shear rheology^5–8^. Prior work has shown that ring polymer melts generally exhibit lower zero-shear viscosities^5,7^ and higher recoverable compliance compared to linear polymer melts^8^. However, even a small amount of linear contaminates alters the rheological response of ring polymer melts, resulting in a drastic increase in viscosity^7,9–11^. Moreover, ring polymer melts were found to show no apparent plateau modulus, but addition of a small fraction of linear chains causes the rubbery plateau to reappear^11^. Therefore, despite many efforts, a central challenge in ring polymer research lies in achieving high purity ring polymer samples that are free of linear chains. Indeed, the ‘purest as-possible’ ring polymer samples prepared by post-polymerization cyclization of linear chains still contain small fractions of linear chains, even after rigorous purification^12^.

From this view, understanding the properties of ring-linear blends is of fundamental interest to elucidate the properties of polymeric samples with mixed chain topology. Prior work has focused on the near-equilibrium properties of ring polymers in ring-linear blends. Computational modeling has shown that ring polymers swell with an increased *R*_*g*_ upon the addition of linear polymers into the background solution^13,14^. Diffusion measurements of ring DNA molecules show that although a ring polymer diffuses approximately 1.4 times faster than a linear chain in a concentrated ring polymer solution, the diffusion coefficient drops dramatically in a background matrix of linear polymers^15–17^. Interestingly, when the background linear chain concentration is well above the overlap concentration c^*^ for linear polymers, a heterogeneous multimode diffusion is observed for rings^18^. Here, it is important to note that c^*^ is defined under equilibrium conditions when the linear polymers are unstretched by flow. Moreover, ring polymers generally exhibit a markedly different molecular weight dependence of the center-of-mass diffusion coefficient compared to linear polymers in background solutions of concentrated linear polymers or melts^19,20^. Nevertheless, it is possible to use single ring polymers as tracer polymers or probes to study entangled linear chain dynamics through small angle neutron scattering (SANS) or neutron spin echo (NSE) spectroscopy^21,22^, essentially by taking advantage of the slow diffusion of rings in ring-linear blends.

The slow-down of ring polymer dynamics in entangled solutions of ring-linear blends has also been theoretically modeled. Here, the stress relaxation mechanism is modeled to include constraint release (CR) of background linear chains^23^ or restricted reptation (RR), where rings relax through amoebae-like conformations in fixed obstacles formed by background linear chains^24^. The once-threaded model (R1) was further proposed to capture ring diffusion along a threaded linear chain^20^. The ‘threading’ phenomenon refers to one or more background linear chains penetrating into an open ring conformation, thereby resulting in a significant decrease in chain diffusion^25,26^. Nevertheless, in reality, the actual diffusion mechanism may rely on a combination of elements from all of these models^25^.

Despite recent progress in understanding the properties of ring-linear blends, a large portion of prior work has focused on the near-equilibrium properties of ring polymers such as equilibrium chain conformation or center-of-mass diffusion coefficient in concentrated solutions or melts. Moreover, ensemble-level experimental methods based on bulk rheology or light scattering provide indirect measurements of the average conformational properties of ring polymers. What are the conformational dynamics of single ring polymers in ring-linear blends under nonequilibrium conditions in flow? Recent advances in single molecule fluorescence microscopy (SMFM) and microscale flow manipulation allow for the direct observation of polymer dynamics under highly nonequilibrium conditions^27^. SMFM can be used to understand phenomena such as dynamic heterogeneity and molecular individualism for polymer chains in flow^28–30^. In recent years, SMFM has been used to study the dynamics of linear polymers in large amplitude oscillatory extensional flow (LAOE)^31,32^, linear polymers in semidilute unentangled solutions^33,34^, and the heterogeneous relaxation dynamics of linear polymers in entangled solutions^35^.

Single molecule techniques have been used to study the flow dynamics of ring polymers, but only in ultra-dilute solutions^36–38^. In the dilute solution regime, it was found that ring polymers show a shifted coil-stretch transition (CST) and less molecular individualism compared to their linear counterparts due to a coupling between intramolecular hydrodynamic interactions (HI) and the constrained chain topology^36,37^. Despite recent work, however, we still lack a comprehensive understanding of the dynamics of ring polymers in non-dilute ring-linear blends.

**Fig. 1:**
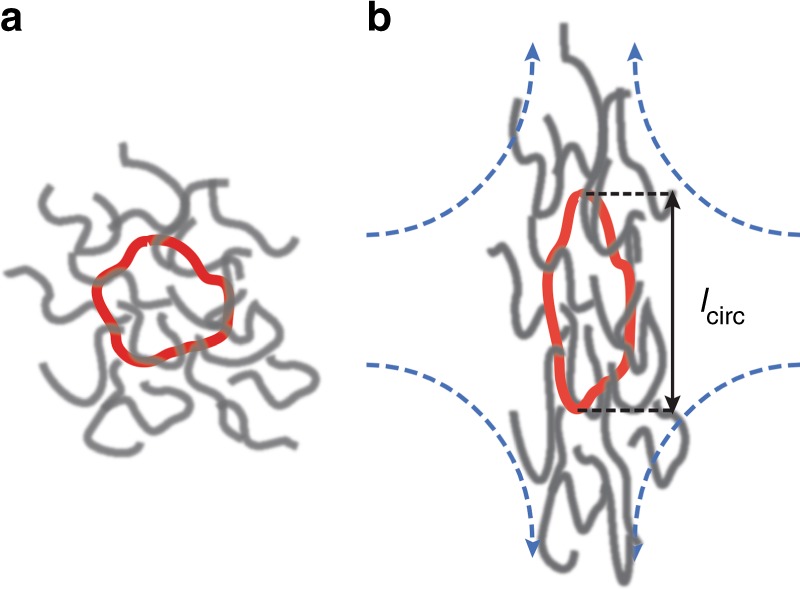
Schematic of the experimental ring-linear system. Fluorescently labeled tracer ring DNA molecules (45 kbp, shown in red) are uniformly dissolved in a background solution of semidilute linear DNA molecules. Dynamics are studied under **a** equilibrium (no flow) conditions and in **b** planar extensional flow. The transient molecular extension of ring polymers *l*_circ_ is directly observed using SMFM

In this work, we study the relaxation dynamics and transient stretching behavior of single ring DNA polymers in background solutions of semidilute unentangled linear DNA (Fig. 1). In particular, fluorescently labeled ring DNA polymers (45 kbp) are uniformly added to background solutions of semidilute linear *λ*-DNA polymers (bacteriophage lambda genomic DNA, 48.5 kbp). Our results show that ring polymers exhibit large conformational fluctuations in extensional flow, even long after the initial transient stretching process has terminated. Remarkably, this behavior occurs even at low background concentrations of linear polymers, down to 0.025 c^*^ based on the linear constituent. These large conformational fluctuations are not observed in the steady-state stretching of linear polymers in the same background solutions^33^, or in the steady-state stretching of ring polymers in dilute solutions (10^−5^ c^*^) under similar flow conditions^36,37^. Based on these findings, we hypothesize that large fluctuations in ring polymer extension arise due to transient threading of linear polymers through partially open rings due to stretched conformations in flow.

## Results

### Longest relaxation time of ring polymers

**Fig. 2:**
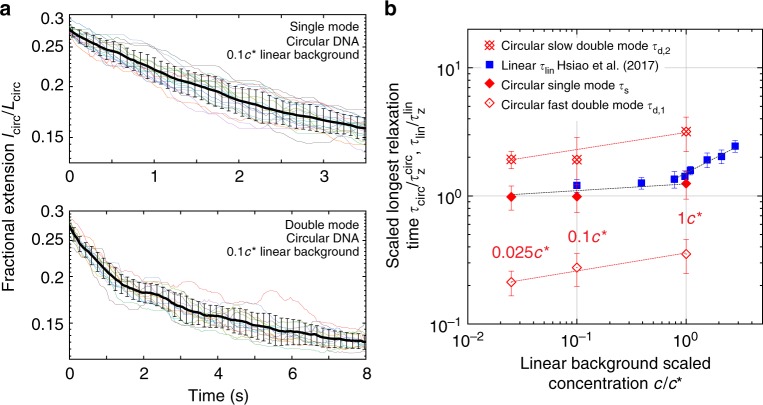
Relaxation of single ring polymers in semidilute linear polymer solutions. **a** Single molecule relaxation trajectories and average relaxation for molecular subpopulations corresponding to (top) single-mode and (bottom) double-mode exponential relaxation trajectories for ring polymers in a background solution of 0.1 c^*^ linear chains. Black curves with error bars (standard deviation) denote the ensemble averaged relaxation trajectory, and the solid thin curves (color) correspond to single molecule relaxation trajectories. Molecular ensembles consist of *n* = 18 molecules for single-mode relaxation and *n* = 13 molecules for double-mode relaxation at 0.1 c^*^. **b** Longest relaxation times (normalized to dilute solution values) for circular (red diamonds) and linear polymers (blue squares) in semidilute unentangled linear solutions as a function of scaled concentration *c*/c^*^. Source data are provided as a Source Data file

**Fig. 3:**
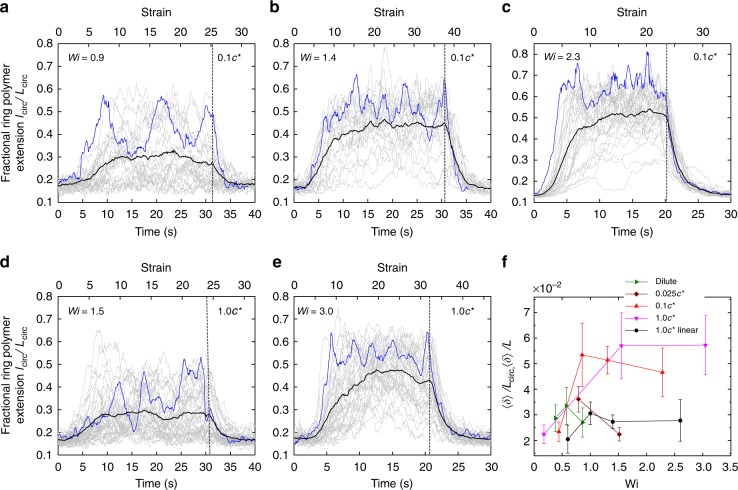
Single molecule trajectories of ring polymers show large conformational fluctuations. Transient fractional extension of ring DNA polymers in semidilute unentangled linear solutions with concentrations of 0.1 c^*^ at **a** *Wi* = 0.9, **b** *Wi* = 1.4, and **c** *Wi* = 2.3, and background linear chain concentration of 1.0 c^*^ at **d** *Wi* = 1.5, and **e**)*Wi* = 3.0. Individual single molecule trajectories are shown in gray lines and ensemble averaged trajectories are shown in a black line. A characteristic individual single molecule trajectory is highlighted in blue line. Molecular ensembles consist of *n* = 24, *n* = 25, and *n* = 33 molecules, respectively for *Wi* = 0.9, 1.4, and 2.3 at 0.1 c^*^, and *n* = 38 for both *Wi* = 1.5 and *Wi* = 3.0 at 1.0 c^*^. The dashed line indicates the time at which the step strain input is stopped. **f** Magnitude of ring polymer conformational fluctuations as a function of *Wi*, plotted as average fractional fluctuation values, such that average chain extension fluctuations 〈*δ*〉 are normalized by the contour length of ring polymers *L*_circ_ and linear polymers *L*, respectively. Data for ring polymers in dilute solution, 0.025 c^*^ linear semidilute solution, 0.1 c^*^ linear semidilute solution, and 1.0 c^*^ linear semidilute solution are denoted as dark cyan right triangle, dark red diamond, red up triangle, and magenta down triangle, respectively. Data for 1.0 c^*^ linear polymers (black circle) are taken from prior work^33^. Error bars represent standard deviation. Source Data are provided as a Source Data file

We began by determining the longest relaxation time of ring polymers in semidilute solutions of linear unentangled polymers ranging in concentration from 0.025 c^*^ to 1.0 c^*^ (Fig. 2). In these experiments, polymer solutions are subjected to a step strain protocol in planar extensional flow at a strain rate $\dot \epsilon$. The accumulated fluid strain (known as the Hencky strain) is given by $\epsilon = {\int}_0^t {\dot \epsilon } dt^\prime$, where *t* is the duration of the step strain rate input. Here, molecular relaxation data was obtained as part of a step strain-relaxation experiment (shown in Fig. 3), such that single chains are exposed to at least $\epsilon \approx 20$ units of strain prior to flow cessation. Prior to chain relaxation, ring polymers are first stretched to high degrees of extension, approximately 60–70% of the fully stretched contour length of the circular chain *L*_circ_, which is taken to be half the contour length of the corresponding linear polymer *L*_lin_. The longest relaxation time of ring polymers is determined by fitting the terminal 30% of the average squared fractional extension (*l*_circ_/*L*_circ_)^2^ to an exponential decay function following the cessation of flow. Here, *l*_circ_ is the maximum fractional extension (end-to-end distance) of the polymers in the two-dimensional flow plane.

Interestingly, our results reveal the emergence of multiple molecular subpopulations for ring polymer relaxation in semidilute solutions. Approximately 75% of the molecular relaxation trajectories are well-described by a single-mode exponential decay (for 0.025, 0.1, and 1.0 c^*^). In addition, a second molecular subpopulation was found to exhibit a double-mode exponential decay (Fig. 2a). Here, the single-mode relaxation time *τ*_s_ is determined from (*l*_circ_/*L*_circ_)^2^ = *A* exp(−*t*/*τ*_s_) + *B*, where *A* and *B* are numerical constants. The fast and slow double-mode relaxation times *τ*_d,1_ and *τ*_d,2_ are determined from (*l*_circ_/*L*_circ_)^2^ = *A*_1_ exp(−*t*/*τ*_d,1_) + *A*_2_ exp(−*t*/*τ*_d,2_) + *B*, where *A*_1_, *A*_2_, and *B* are numerical constants. In prior single molecule studies of linear polymer relaxation, Zhou et al.^35^ observed a molecular subpopulation exhibiting double-mode exponential relaxation behavior, but only in entangled solutions of linear polymers. Interestingly, here we observe multiple molecular subpopulations with single and double-mode relaxation behavior for ring polymers in background solutions of semidilute unentangled linear chains. This behavior is qualitatively different than the relaxation of linear polymers in ultra-dilute solutions (10^−5^ c^*^)^27^, linear polymers in semidilute unentangled solutions^33^, and ring polymers in ultra-dilute solutions (10^−5^ c^*^)^36,37^.

The longest relaxation times of ring and linear polymers as a function of background concentration of linear polymers is plotted in Fig. 2b. To enable direct comparison with linear chain relaxation across a wide range of concentrations, relaxation times for ring polymers *τ*_*s*_, *τ*_d,1_ and *τ*_d,2_, and linear polymers *τ*_lin_ are normalized by their corresponding longest relaxation times in ultra-dilute solutions (10^−5^ c^*^), denoted as *τ*_circ,z_, and *τ*_lin,z_, respectively. Absolute values of relaxation times are shown in Supplementary Table 1. In dilute solutions, ring polymers were observed to relax approximately three times faster than linear polymers^36^. This relation appears to approximately hold for the single-mode relaxation time *τ*_s_ for rings in semidilute linear background solutions. However, the slow double-mode relaxation time *τ*_d,2_ for rings is approximately two times smaller than the longest relaxation time for linear chains, and the fast double-mode relaxation time *τ*_d,1_ for rings is substantially smaller (factor of ~15) compared to linear chains at the same background solution concentrations (Supplementary Table 1). As shown in Fig. 2b, as the concentration of the background linear polymer solution is increased, the single-mode relaxation time for rings slightly increases with a power law exponent of 0.07 ± 0.02 compared to 0.04 ± 0.02 for pure linear polymers. The slow double-mode relaxation time *τ*_d,2_ scales with concentration with a power-law exponent of 0.13 ± 0.03, and the fast double-mode relaxation scales with concentration with a similar power-law exponent of 0.14 ± 0.02.

These results show clear differences for ring and linear chain topologies in non-dilute polymer solutions. For purely linear polymer solutions with homogeneous chain topology, longest relaxation times are described by a weak power-law dependence as a function of concentration for *c* < c^*^, which is consistent with prior single molecule experiments^33^. On the other hand, ring polymers exhibit two molecular subpopulations when relaxing in semidilute solutions of linear chains, even at low concentrations $c \ll c^ \ast$. In particular, the single-mode relaxation time for rings in semidilute linear solutions appears to be consistent with the power-law scaling relation for linear chains, but we further observe a second molecular subpopulation described by double-mode exponential relaxation. This behavior is analogous to the relaxation behavior for homogeneous linear solutions at higher concentrations in the entangled regime for *c* > *c*_e_, where *c*_e_ is the critical entanglement concentration^35^. Taken together, these results highlight the importance of chain architecture on the relaxation behavior of polymers in non-dilute solutions.

### Transient stretching dynamics of ring polymers

We next investigated the transient stretching dynamics of ring polymers in background solutions of semidilute unentangled linear polymers at concentrations of 0.025 c^*^, 0.1 c^*^, and 1.0 c^*^ (Fig. 3). The flow strength is characterized by the Weissenberg number $Wi = \dot \epsilon \tau _{\mathrm{s}}$, where *τ*_s_ is the longest average single-mode relaxation time for a ring polymer in the corresponding background solution concentration. In these experiments, ring polymers are first allowed to relax to an equilibrium conformation for a duration of ≥2*τ*_s_ under zero flow conditions. At time *t* = 0, the solution is exposed to a step strain rate input for a precisely controlled amount of fluid strain $\epsilon$. During the step strain deformation event, individual ring polymers are confined near the stagnation point in planar extensional flow using a feedback-controlled flow device known as a Stokes trap^31,39^. In this way, single polymers are confined for long times in extensional flow with minor variations to the inlet flow rates such that the strain rate is constant, as discussed in prior work^31,39^. Following the step strain deformation, the flow is stopped and ring polymers relax back to thermal equilibrium, resulting in a random coil conformation.

Transient fractional extension *l*_circ_/*L*_circ_ for single ring polymers is shown in Fig. 3a–e for 0.1 c^*^ and 1.0 c^*^ solutions (results for 0.025 c^*^ shown in Supplementary Fig. 1). In all cases, ring polymers are exposed to an accumulated fluid strain $\epsilon \ge 25$, and at least 40 single molecule trajectories are analyzed. The ensemble-average fractional extension overall individual trajectories is shown in black, and one representative single molecule trajectory in each plot is highlighted in blue (Fig. 3a–e). Individual ring polymers show a broad variability in transient stretching dynamics. Remarkably, large chain conformational fluctuations are observed at all *Wi* and for all background solution concentrations at 0.025 c^*^, 0.1 c^*^, and 1.0 c^*^. Here, dramatic fluctuations in ring extension are observed long after the initial transient stretching process has passed, with large conformational fluctuations continuing well beyond accumulated fluid strains $\epsilon \ge 8$. We note that such large magnitude conformational fluctuations are not observed for ring polymers in dilute solution^36^ or for pure linear polymers in semidilute solution^33^. This dynamic behavior suggests that ring polymers do not reach a true steady-state chain extension in extensional flows of semidilute solutions, unlike pure solutions of linear chains in the semidilute regime, where single polymers are observed to reach a steady-state extension in extensional flow^33^. Interestingly, large conformational fluctuations are observed for isolated linear polymers in shear flow, albeit with a completely different physical origin due the rotational flow component coupled with the extensional component in simple shear flow^29^.

The magnitude of chain extension fluctuations is quantified in terms of an average fluctuation in fractional chain extension 〈*δ*〉/*L*_circ_ (Methods). In brief, this quantity provides a measure of the magnitude of conformational extension fluctuations for a single chain after the initial transient stretching phase in extensional flow, averaged over the molecular ensemble for a given *Wi* and background solution concentration. Chain extension fluctuations are calculated for rings 〈*δ*〉/*L*_circ_ and compared to fluctuations 〈*δ*〉/*L* for pure linear semidilute solutions, as shown in Fig. 3f. Chain extension fluctuations increase with increasing concentration of background linear chains from the ultra-dilute regime dilute 10^−5^ c^*^ to 1 c^*^. Importantly, ring polymers show large conformational fluctuations in semidilute linear solutions, and the magnitude of chain extension fluctuations significantly increases near the coil-stretch transition (CST), corresponding to *Wi* ≈ 0.5. On the other hand, relatively small chain fluctuations are observed in pure linear solutions even at 1 c^*^. This supports the observation that chain conformational fluctuations is unique to ring polymers, which is likely due to the threading of linear chains into rings, even at low concentrations of background linear chains such that $c \ll c^ \ast$.

**Fig. 4:**
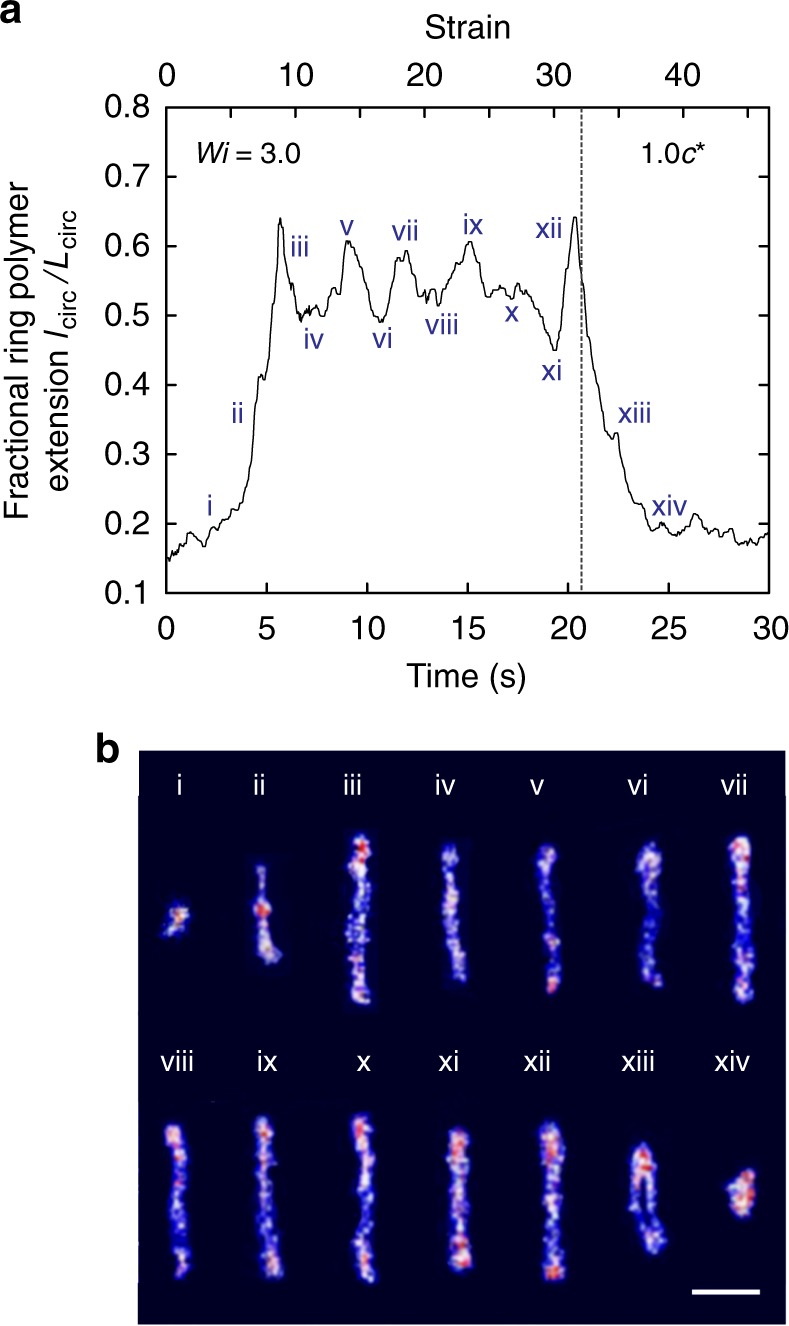
Characteristic transient stretching trajectory and single molecule snapshots. Experimental data shows large magnitude extension fluctuations for ring polymers in extensional flow. **a** Representative single molecule trajectory for a ring polymer in 1.0 c^*^ semidilute unentangled linear polymer solution at *Wi* = 3.0. The characteristic timescale between each peak is 2.8 *s* corresponding to 4.4 strain units. Source data are provided as a Source Data file. **b** Single molecule snapshots of the ring polymer corresponding to the trajectory in **a**, where the Roman numerals correspond to individual time points along the trajectory. Scale bar = 2.5 μm

A characteristic dynamic trajectory for a single ring polymer in a 1.0 c^*^ semidilute linear solution at *Wi* = 3.0 is shown in Fig. 4a. Corresponding single polymer snapshots are shown in Fig. 4b, where Roman numerals denote the peak and trough positions on the single molecule trajectory in Fig. 4a. From a qualitative perspective, dynamic stretching trajectories of single ring polymers appear to show an apparent characteristic periodic frequency in chain extension fluctuations. In particular, the characteristic timescale for extension fluctuations can be quantified by determining the autocorrelation function and power spectral density (Supplementary Fig. 3). The timescale is found to be 2.8 s (corresponding to 4.4 strain units) for the trajectory in Fig. 4a. These effects are further quantified and analyzed for additional molecular trajectories, as discussed below.

We conjecture that large magnitude chain extension fluctuations arise due to transient threading of linear polymers in the background solution into partially stretched, open conformation ring polymers. The transport of linear chains into open ring polymers partially stretched by flow leads to repeated and continuous hooking and unhooking events of linear chains threading into ring polymer backbones, which leads to repeated cycles of transient chain extension (hooking) and retraction (unhooking) for a given ring polymer in extensional flow. From this view, threading events are highly dynamic in the presence of fluid flow.

### Probability distributions of transient chain stretching

**Fig. 5:**
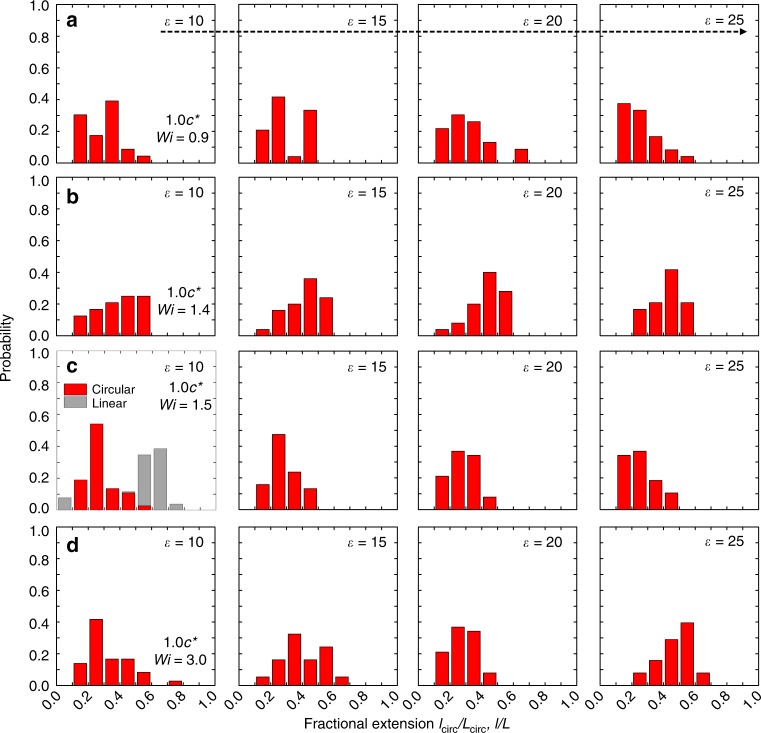
Probability distribution of ring polymer extension. Experimental data show ring polymers (red bars) in background solutions of semidilute linear polymers at concentrations of 0.1 c^*^ and 1.0 c^*^ and accumulated fluid strains of $\epsilon$ = 10, $\epsilon$ = 15, $\epsilon$ = 20, $\epsilon$ = 25. Molecular ensembles consist of **a** *n* = 24, **b** *n* = 25, **c** *n* = 38, and **d** *n* = 38 molecules. Data for 1.0 c^*^ linear polymers (gray bars) are from Hsiao et al.^33^. Source data are provided as a Source Data file

We further characterized the probability distribution of ring polymer extension in flowing solutions of semidilute linear chains (Fig. 5). Here, we consider chain extension distributions for accumulated fluid strain $\epsilon > 10$, focusing only on ring polymer dynamics long after the initial transient stretching phase has ended. A broad distribution of polymer fractional extension is observed for ring polymers in semidilute linear solutions in extensional flow. Importantly, these observations greatly contrast with the behavior of pure homogeneous linear polymers. For example, most linear polymers are stretched to fractional extensions *l*_lin_/*L* > 0.6 in 1.0 c^*^ linear background solutions at *Wi* = 1.0, whereas ring polymers exhibit a broad distribution in fractional extension centered around a fractional chain extension of *l*_circ_/*L*_circ_ ≈ 0.3 (Fig. 5c). The probability distribution of ring polymer extension shifts slightly to higher fractional extensions upon increasing the accumulated fluid strain, but the distributions in ring polymer extension remain noticeably broad. We attribute the broad distributions in ring polymer extension to the effect of dynamical threading facilitated by flowing linear polymer chains, which results in large magnitude chain extension fluctuations and broad probability distributions of extension.

### Autocorrelation of chain fluctuations

**Fig. 6:**
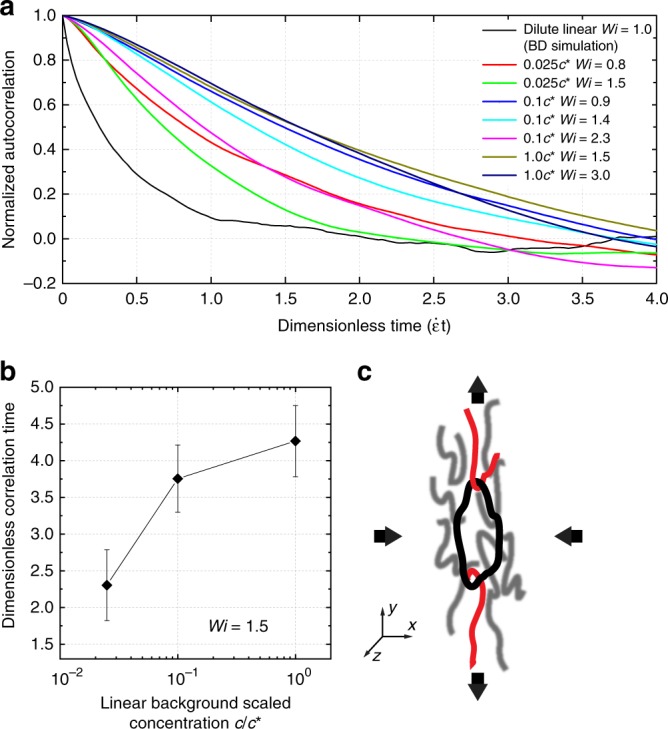
Quantitative analysis of ring polymer conformational fluctuations. **a** Autocorrelation of fluctuations in ring polymer extension as a function of *Wi* and background concentration after the initial transient phase. Molecular ensembles consist of *n* = 200 molecules for dilute linear chains (black solid line); *n* = 32 and *n* = 31 for *Wi* = 0.8 (red solid line) and *Wi* = 1.5 (green solid line) at 0.025 c^*^; *n* = 24, *n* = 25, and *n* = 33 for *Wi* = 0.9 (blue solid line), *Wi* = 1.4 (cyan solid line), and *Wi* = 2.3 (magenta solid line) at 0.1 c^*^; *n* = 38 for *Wi* = 1.5 (dark yellow solid line) and *Wi* = 3.0 (navy solid line) at 1.0 c^*^. **b** Characteristic correlation time (black diamond) of ring polymer fluctuations at *Wi* = 1.5 as a function of background solution concentration. Error bars represent standard deviation. Molecular ensembles consist of *n* = 31, *n* = 25, *n* = 38 molecules, respectively from 0.025 c^*^ to 1.0 c^*^. **c** Schematic of two linear polymers threading into a ring polymer in planar extensional flow. Source Data are provided as a Source Data file

We analyzed the transient conformational fluctuations of ring polymers in flow by determining the autocorrelation of chain extension fluctuations (Methods). In brief, the autocorrelation for chain extension fluctuations was determined based on the instantaneous value of chain extension relative to the mean extension after the initial transient stretching phase in extensional flow. Ensemble-averaged autocorrelation functions for ring extension fluctuations as a function of *Wi* and solution concentration are shown in Fig. 6a. Our results show that the conformational fluctuations of rings exhibit a slower decay and increased correlation time as opposed to a sharp decay occurring over randomly distributed timescales. Upon decreasing *Wi* at a constant background concentration (Fig. 6a), or increasing background polymer concentration at constant *Wi* (Fig. 6b), the autocorrelation function decays with a longer timescale. This indicates that in an average sense, the probability of interchain interactions increases with increased concentration of linear chains in the background solution. These results are consistent with the general trends in the magnitude of the average ring extension fluctuation, as shown in Fig. 3f. The correlation time for threading fluctuations ranges between 2 and 5 strain units for different values of *Wi* and background concentration.

Interestingly, the ensemble-averaged autocorrelation functions shown in Fig. 6a do not reflect the apparent regular periodic nature exhibited the characteristic molecular trajectory shown in Fig. 4a. For any molecular-level process, one would expect a distribution of dynamic behavior across the molecular ensemble due to the role of stochasticity. With this in mind, it is likely that one subpopulation of ring polymers will exhibit fairly regular threading/unthreading events, but other subpopulations might show intermittent threading. In other words, conformational fluctuations can be more irregular during the limited amount of strain that we impose on (and observe over) for the tracer ring polymers. For example, a linear chain could thread and unthread over distributed timescales, which could in some cases be followed with a long time before the next threading occurs, depending on the background polymer concentration. In this case, the autocorrelation function for this class of molecular trajectories will not be perfectly periodic, which results in a loss of periodicity in the ensemble-averaged autocorrelation functions.

### Average unsteady stretch of ring polymers

**Fig. 7:**
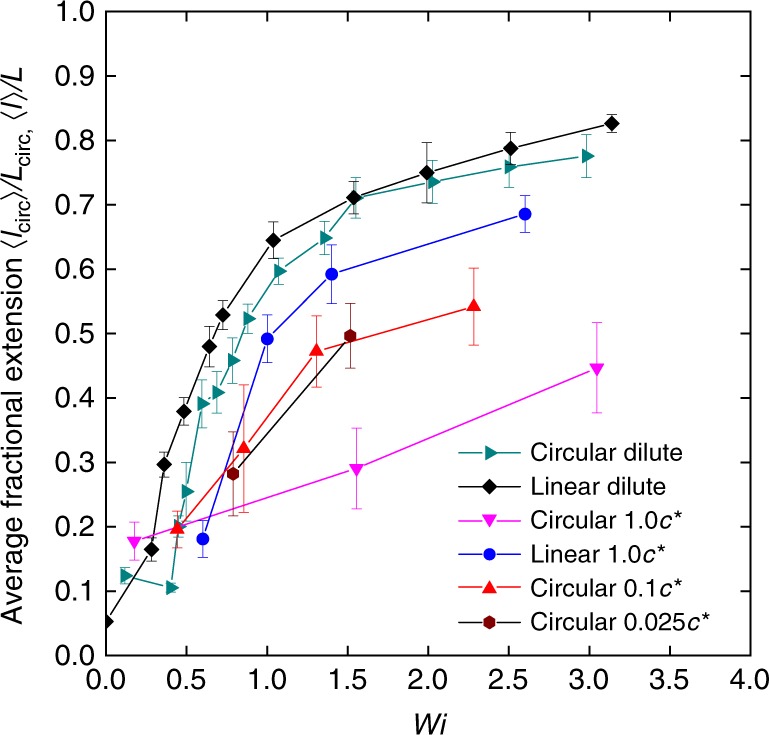
Steady and average unsteady fractional extension of linear and ring polymers in extensional flow. Experimental data for ring polymers in semidilute unentangled linear solutions at 0.025 c^*^, 0.1 c^*^, and 1.0 c^*^ are shown in dark red hexagon, red up triangle, and magenta down triangle, respectively. Experimental data for dilute ring polymers (dark cyan right triangle) are taken from Li et al.^36^, and data for 1.0 c^*^ linear polymers (blue circle) are taken from Hsiao et al.^33^. Molecular ensembles consist of at least *n* ≥ 35 molecules at each concentration and error bars represent standard deviation. Source data are provided as a Source Data file

Based on these results, it is clear that ring polymers exhibit large conformational fluctuations in semidilute solutions, even long after the initial transient stretching phase has ended. We determined an average unsteady fractional extension for ring polymers in extensional flow (Fig. 7), analogous to a method used in determining average unsteady polymer stretch in large amplitude oscillatory extension^31,32^. Here, we define average unsteady chain extension as the average value of fractional extension for accumulated fluid strains $\epsilon > 10$, again focusing on ring polymer dynamics long after the initial transients have died out. For direct comparison, we also plot the average steady fractional extension for linear polymers in dilute solution^33,36^, ring polymers in dilute solution^36^, and semidilute pure solutions of linear polymers at 1.0 c^*^ in extensional flow (Fig. 6)^33^. Unlike linear polymers, ring polymers in dilute solutions show a slightly shifted coil-stretch transition (CST) due to intramolecular hydrodynamic interactions (HI) between the two strands under planar extensional flow^36,37^. Prior work also found that semidilute solutions of pure linear chains solutions exhibit slightly inhibited steady fractional extensions and a larger critical Weissenberg number at the CST (defined as *Wi*_c_), which is approximately 1.6 times larger than that for linear polymers in dilute solutions^33^.

Interestingly, our results show that the average unsteady fractional extension for ring polymers in semidilute linear solutions is significantly delayed with a marked increase in *Wi*_c_ at the CST (Fig. 7). We can estimate *Wi*_c_ for ring polymers in semidilute solutions based on the average fractional extension at CST, given by $\langle \tilde l_{\mathrm{c}}\rangle = \langle l_{\mathrm{c}}\rangle /L_{{\mathrm{circ}}}$, which is calculated in a logarithmic scale between the coiled and stretched states such that $ln\left\langle {\tilde l_{\mathrm{c}}} \right\rangle ^2 = \left( {ln\left\langle {\tilde l_0} \right\rangle ^2 + ln\left\langle {\tilde l_{{\mathrm{max}}}} \right\rangle ^2} \right)/2$^33,40^. Here, $\tilde l_0 = \langle l_0\rangle /L_{{\mathrm{circ}}}$ is the equilibrium fractional extension of ring polymers and $\tilde l_{{\mathrm{max}}} = \langle l_{{\mathrm{max}}}\rangle /L_{{\mathrm{circ}}}$ is the maximum fractional extension observed for ring polymers in our experiments. In this way, the critical Weissenberg number at the CST for ring polymers is found to be *Wi*_c_ = 0.8, *Wi*_c_ = 0.9, and *Wi*_c_ = 1.5 in semidilute linear solutions with concentrations of 0.025 c^*^, 0.1 c^*^, and 1.0 c^*^, respectively. Clearly, polymer architecture and concentration impact the dynamics of ring polymers in extension flow, thereby influencing both the critical Weissenberg number at the CST and the average unsteady stretch of polymers in flow.

## Discussion

Understanding the effect of molecular architecture on the dynamics of polymers with different chain topologies is an intriguing problem in soft materials. In the context of linear-ring blends, prior work has focused on the near-equilibrium properties of ring polymers in concentrated solutions or melts, but less is understood about the nonequilibrium dynamics of these materials. In this work, we use single molecule methods to study the dynamics of ring polymers in background solutions of semidilute linear polymers. These experiments reveal large magnitude extension fluctuations of rings in non-dilute solutions. In prior studies, the proposed mechanism of chain threading has been deduced from indirect measurements based on increased zero-shear viscosity^6^, changes in the plateau modulus^11^, or reduced diffusion coefficients^20^. Our work, however, provides molecular-level evidence of chain threading into open conformations of ring polymers in flow.

Remarkably, our results show that fluctuations in ring polymer extension occur in background solutions of linear chains at concentrations as low as 0.025 c^*^, which is the lowest concentration tested in this work. These results suggest that even a small amount of linear chains can be transported through ring polymers in extensional flow, thereby leading to fluctuations in chain extension. On the other hand, evidence of chain threading at equilibrium (no flow) is typically observed only at much higher solution concentrations^41^, in stark contrast with our findings under nonequilibrium conditions. It is known that anisotropy in molecular extension due to flow can lead to an increase in local concentration^42^. For instance, the overlap concentration c^*^ is defined as c^*^ = *M*/[(4*π*/3)*R*^3^*N*_*A*_], where *M* is the molecular weight and *R* is the size of the molecule. At equilibrium, *R* is taken as the radius of gyration for the molecule such that *R* = *R*_*g*_. However, under nonequilibrium flow conditions, the spatial extent (or average molecular size) of linear chains in the background are much larger than the equilibrium coil size. Given an increase in molecular size due to stretching in flow, the overlap concentration in flow can be surprisingly smaller than that at equilibrium, as c^*^ scales with chain size such that c^*^ ~ *R*^−3^^43^. Assuming that linear DNA polymers in the background solution are stretched to approximately 30% of the polymer contour length in extensional flow, a solution concentration of 0.025 c^*^ at equilibrium corresponds to approximately 5 c^*^ in extensional flow, which could conceivably result in appreciable intermolecular interactions and polymer chain threading. These arguments can be further rationalized by considering intermolecular distances in semidilute solutions. At equilibrium, linear *λ*-DNA molecules can be modeled as uniformly dispersed spherical particles, yielding a mean shortest distance between particles of approximately 0.5–2 μm at 0.025 c^*^ (1.2 μg mL^−1^), assuming *R*_*g*_ ≈ 0.7 μm^44,45^. On the other hand, if the background linear DNA molecules are stretched to 30% of their contour length in extensional flow, the radius-of-gyration of the linear chains increases by a factor of 4^46^. In this way, the process of polymer stretching in flow leads to an increase in particle size greater than the interparticle distance, thereby causing the linear chains to overlap. To this end, observation of ring polymer fluctuations provides further evidence that only a small fraction of linear chains is required to alter the rheological response of ring polymers^7,9,11,12,47^.

The dynamic behavior of ring polymers in semidilute linear solutions starkly contrasts with the dynamics of ring or polymers in ultra-dilute solution extensional flows, where the extension fluctuations are generally largest near the coil-stretch transition. In particular, the magnitude of ring polymer extension fluctuations increases with increasing concentration of linear polymers in the background solution (Fig. 3f). Moreover, chain fluctuations drastically increase when the flow strength is above the critical *Wi* at the coil-stretch transition, when most of the ring polymers are appreciably stretched. Upon increasing *Wi*, the magnitude of chain extension fluctuations eventually plateaus for both ring and linear polymers in semidilute solutions, despite large differences in the fluctuation magnitude.

Based on these results, we hypothesize that ring polymers undergo a dynamic chain threading mechanism as illustrated in Fig. 6c. As ring polymers are stretched in extensional flow, the circular topology opens up, which allows for linear polymer chains in the background solution to be transported into open rings, thereby resulting in transient threading events. In turn, this dynamic behavior results in transient hooked structures of linear chains, as shown in Fig. 6c. Such conformations result in enhanced frictional drag due to the threaded linear chains. Moreover, the hooked conformation of linear chains is known to be fairly sluggish to unravel in extensional flow^27^, which provides a finite time over which the hydrodynamic drag on the ring-linear polymer structure is increased, thereby resulting in ring polymer stretch to extensions larger than the mean value. On the other hand, when the linear chains finally unhook from ring chain, the enhanced hydrodynamic drag instantaneously vanishes, resulting in a decrease in fractional extension. During this process, linear chains are continually transported into open ring polymers, which results in repeated hooking and unhooking events with associated fluctuations in chain extension. However, it is important to note that the threaded states are not necessarily limited to a doubly-threaded state as illustrated in Fig. 6c. It is possible that multiple simultaneous hooking and unhooking events could result in either singly-threaded as well as multiply-threaded states^25^.

Our results further reveal the existence of two molecular subpopulations for ring relaxation. One subpopulation exhibits single-mode relaxation behavior, which we attribute to ring polymers that are not associated with a threaded linear polymer. On the other hand, the emergence of a second molecular subpopulation with double-mode relaxation behavior for ring polymers suspended in a solution of semidilute linear chains likely results from interactions with threaded linear chains. The occurrence of double-mode relaxation behavior increases upon increasing background linear chain concentration, indicating higher probabilities of interactions with threaded linear chains (Supplementary Fig. 4). Prior to flow cessation, our results suggest that a large fraction of ring polymers have at least one threaded linear polymer. Immediately following cessation of flow, a hooked linear chain is able to quickly retract along with the partner ring polymer, which could account for the fast double-mode timescale. Moreover, the slow double-mode timescale is longer than the single-mode relaxation time for rings, which is suggestive of complex ring-linear threading interactions during terminal relaxation.

In this work, we directly observe the dynamics of ring polymers in semidilute linear polymer solutions using single molecule fluorescence microscopy. Ring polymers exhibit broad distributions in extension as a function of accumulated fluid strain, which contrasts with the behavior of their linear counterparts. We attribute large conformational chain fluctuations to the transient threading of linear polymers into open ring conformations in flow. Quantitative analysis of ring fluctuations reveals a characteristic frequency or timescale associated with extension fluctuations, which is analyzed as a function of *Wi* and concentration of polymer chains in the background solution. Based on these results, we propose a transient hooking mechanism for linear polymer threading into ring polymers in flow. In this way, the continuous hooking and unhooking of linear chains leads to the significant chain fluctuations in ring polymers in flow. From a broad view, our results provide key molecular insights into the dynamics of ring polymers in ring-linear blends, which is a major stride forward in our understanding of non-dilute polymer solutions with mixed ring/linear topologies. Future investigations focusing on the flow dynamics of ring-linear blends as a function of the molecular architecture and concentration will further elucidate the physics and properties of these intriguing systems.

## Methods

### Preparation of 45 kbp ring DNA

Double-stranded DNA constructs 45 kbp in length are prepared via replication of fosmids in *Escherichia Coli*, followed by extraction and purification. In brief, the DNA constructs are extracted from cultures of *Escherichia Coli* cells by alkaline lysis followed by treatment of an acidic detergent solution such that the cloned DNA is renatured. The genomic DNA and cellular debris precipitate are removed by centrifugation. Supercoiled DNA constructs are then converted to relaxed circular conformation via treatment with Topoisomerase-I (New England Biolabs)^15^. To remove any contaminating RNA, the sample is treated with RNase A, and protein is removed by phenol-chloroform extraction followed by dialysis. The sample is finally concentrated by a second isopropanol precipitation and its topology and concentration is determined using gel electrophoresis^15,48^.

### Preparation of semidilute unentangled linear solutions

For all experiments, bacteriophage *λ*-phage DNA (New England Biolabs, 48.5 kbp, 31.5 × 10^6^ Da) in a buffered aqueous solution with 10 mM Tris/Tris-HCl (pH 7.4) and 1 mM ethylenediaminetetraacetic acid (EDTA) is used to prepare background semidilute unentangled solution. The stock *λ*-DNA solution is typically provided in a concentration of 500 μg mL^−1^ (≈10 c^*^). To prepare semidilute unentangled *λ*-DNA solutions at different concentrations, the stock *λ*-DNA concentration is first measured using a UV-vis spectrophotometer (Nanodrop 1000, Thermo Fisher) at a wavelength of 260 nm and an extinction coefficient $\epsilon$ = 0.02 mL μg^−1^ cm^−1^. The stock *λ*-DNA concentration is generally between 300–500 μg mL^−1^ for different batches. We then calculate the mass of DNA required at different target concentrations with a desired volume of 5 mL, which is a typical sample volume used for single molecule imaging and viscosity measurements. Next, based on the stock *λ*-DNA concentration, a working volume of *λ*-DNA solution containing the desired concentration is prepared and heated to 65 °C for 10 min followed by snap cooling to 0 °C to minimize formation of DNA concatemers. For all polymer concentrations, the stock *λ*-DNA working volume is concentrated to ~50 μL using a MiVac Quattro concentrator (Genevac, UK) and viscous buffer solution containing 30 mM Tris/Tris-HCl (pH 8.0), 2 mM EDTA, 5 mM NaCl and sucrose (65% w/w) is added to the working volume to 5 mL. Prepared semidilute unentangled *λ*-DNA solutions then undergo a series of repeated heating and mixing cycles to ensure sample homogeneity. Samples are first heated to 55 °C for 5 min and mixed (by gentle rotation) for 10 min at room temperature (20 °C). This cycle is repeated for twice followed by an overnight gentle rotational mixing procedure at 4 °C. The concentration of the semidilute unentangled *λ*-DNA solution is determined before each single molecule experiment using a UV-vis spectrophotometer. For *λ*-DNA molecules, the overlap concentration c^*^ = 50 μg mL^−1^ at 22.5 °C. The final sample concentrations (in units of μg mL^−1^) and corresponding scaled concentrations in units of c^*^ are tabulated in Table 1.

**Table 1:** Concentration of *λ*-DNA solutions used in this work

Concentration (c^*^)	0.025	0.1	1.0
Concentration (μg mL^−1^)	1.2	5.0	50

### Fluorescent labeling of 45 kbp ring DNA

For single molecule imaging, small amounts of 45 kbp circular DNA molecules are fluorescently labeled with an intercalating dye (YOYO-1, Molecular Probes, Thermo Fisher) with a dye-to-base pair ratio of 1:4 for >1 h in dark at room temperature. Trace amounts of fluorescently labeled 45 kbp DNA are then added to background solutions of unlabeled semidilute unentangled *λ*-DNA, resulting in a final labeled DNA concentration of 2 × 10^−3^ μg mL^−1^ (~10^−5^c^*^). In addition, a small amount of reducing agent *β*-mercaptoethanol (14 μM) and coupled enzymatic oxygen scavenging system containing glucose (50 μg mL^−1^), glucose oxidase (0.01 μg mL^−1^), and catalase (0.004 μg mL^−1^) are added into the entangled solution to suppress photobleaching and photocleaving of fluorescently labeled 45 kbp DNA molecules. The semidilute unentangled solution mixture is rotationally mixed for >20 min before single molecule imaging. All the solution viscosity *η*_s_ is determined using a cone and plate viscometer (Brookfield, USA) at 22.5 °C.

### Optics and imaging

Single molecule fluorescence microscopy and imaging is performed using an inverted epifluorescence microscope (IX71, Olympus) coupled to an electron-multiplying charge coupled device (EMCCD) camera (iXon, Andor Technology). Labeled DNA solutions are illuminated using a 50 mW 488 nm laser (Spectra-Physics, CA, USA) directed through a 2.2 neutral density filter (ThorLabs, NJ, USA), a 488 nm single-edge dichroic mirror (ZT488rdc, Chroma). Fluorescence emission is collected by a 1.45 NA, ×100 oil immersion objective lens (UPlanSApo, Olympus), and a 525 nm single-band bandpass filter (FF03-525/50-25, Semrock) is used in the detection path. Finally, images are acquired by an Andor iXon EMCCD camera (512 × 512 pixels, 16 μm pixel size) under frame transfer mode at a frame rate of 33 Hz (0.030 s^−1^). Experimental images obtained using fluorescence microscopy are analyzed using an in-house Matlab code based on algorithms reported in the literature^49,50^. The Kuhn step size of fluorescently labeled DNA is taken to be 0.132 μm^27^, and the contour length of fluorescently labeled *λ*-DNA is approximately 21.2 μm. Also, the contour length of the full circular topology of fluorescently labeled Fos 45 DNA is approximately 20 μm, with the fully stretched length of the Fos 45 ring polymer equal to *L*_circ_ = 10 μm.

### Microfluidic device and flow field characterization

Two-layer PDMS-based microfluidic devices are fabricated using standard techniques in soft lithography (Supplementary Fig. 2)^32^. In brief, the microfluidic device contains a fluidic layer situated below a control layer containing a fluidic valve. The fluidic layer is fabricated to contain a cross-slot channel geometry (300 μm width by 100 μm height) to generate planar extensional flow, and the control layer contains a pressure-driven valve to control fluid flow. Strain rate and flow field characterization are performed using particle tracking velocimetry (PTV)^31^.

### Average chain extension fluctuations

The average fractional chain extension fluctuation quantity 〈*δ*〉/*L*_circ_ is defined as:

1 $$\frac{{\langle \delta \rangle }}{{L_{{\mathrm{circ}}}}} = \frac{{\mathop {\sum}\nolimits_{n = 1}^N {\sqrt {\mathop {\sum}\nolimits_{t_{90}}^{t_f} {[l_n(t) - \langle l_n\rangle ]^2} } } }}{{NL_{{\mathrm{circ}}}}}$$

where *l*_*n*_(*t*) denotes the instantaneous polymer extension, 〈*l*_*n*_〉 denotes the time-averaged or mean polymer extension, and *N* is the total number of individual trajectories in the ensemble. Here, *t*_f_ is the time at which the step strain rate input is halted, corresponding to the instant at which the ring polymer just begins to relax as indicated by the dashed line in Fig. 3a–e, and *t*_90_ is defined as the time at which the fractional polymer extension first reaches 90% of the average fractional extension at *t*_f_. Experimental data points are discrete points with a frame rate 33 Hz, hence a summation is used instead of an integral for continuous quantities. In this way, the initial transient stretching of ring polymers is discarded, and we compute the chain extension fluctuation quantities only after the initial transient phase has died out.

### Autocorrelation of chain extension fluctuations

The autocorrelation function of a real-valued, integrable fluctuating quantity *x*(*t*) is defined as:

2 $$C_{x,x}(\lambda ) = \langle x(t)x(t + \lambda )\rangle _t$$

where *λ* denotes the offset time, and 〈⋅〉_*t*_ denotes a time-averaged quantity. To quantitatively understand the threading dynamics the linear chains into the rings, we determined the autocorrelation function for chain extension fluctuations. Here, we define fluctuations in projected extensions as the mean extension 〈*l*〉_*t*_ subtracted from the instantaneous chain extension *l*(*t*), such that: *l*′(*t*) = *l*(*t*) − 〈*l*〉_*t*_. The autocorrelation function $C_{l^\prime ,l^\prime }$ is thus given by:

3 $$C_{l^\prime ,l^\prime }(\lambda ) \equiv \frac{{\langle l^\prime (t)l^\prime (t + \lambda )\rangle _t}}{{\langle {l^\prime}^ 2(t)\rangle _t}} = \frac{{{\int}_{ - \infty }^\infty {l^\prime (t)l^\prime (t + \lambda )dt} }}{{{\int}_{ - \infty }^\infty {{l^{\prime}}^2(t)dt} }}$$

where *C*_*l*′,*l*′_ is normalized by the autocorrelation function at zero offset time *λ* = 0. When calculating the autocorrelation function, the initial transient stretching phase is discarded based on the same criterion as the averaged fractional fluctuation quantity such that only the fractional chain extension from *t*_90_ to *t*_f_ is considered. Finally, the correlation time is determined based on the time at which the autocorrelation function reaches zero and is normalized by the fluid strain rate $\dot \epsilon$.

### Reporting summary

Further information on experimental design is available in the Nature Research Reporting Summary linked to this article.

## Supplementary information

Supplementary Information

Reporting Summary

Source Data

## Data Availability

The source data underlying Figs 2, 3, 4a, 5, 6a, b and 7 and Supplementary Figs 1, 3 and 4 are provided as a Source Data file.
