# Supplementary material for: Effect of molecular architecture on ring polymer dynamics in semidilute linear polymer solutions
Source: Nat Commun. 2019 Apr 15;10:1753. doi: 10.1038/s41467-019-09627-7 (PMC6465312; doi:10.1038/s41467-019-09627-7)
Supplement: Supplementary file 1 — Supplementary Information [file 41467_2019_9627_MOESM1_ESM.pdf]

Supplementary Information: Effect of Molecular Architecture on Ring Polymer Dynamics in Semidilute Linear Polymer Solutions

Charles M. Schroeder, et al. *[1,2,3]

[1]Department of Materials Science and Engineering, University of Illinois at Urbana-Champaign, Urbana, Illinois 61820, USA
[2]Beckman Institute for Advanced Science and Technology, University of Illinois at Urbana-Champaign, Urbana, Illinois 61801, USA
[3]Department of Chemical and Biomolecular Engineering, University of Illinois at Urbana-Champaign, Urbana, Illinois 61801, USA

*To whom correspondence should be addressed: cms@illinois.edu

Supplementary Table 1. Characteristic relaxation times for circular and linear DNA molecules in linear semidilute unentangled solutions. The relaxation times are scaled with the solvent viscosity of the background linear semidilute unentangled solutions. Concentration is scaled to the overlap concentration c^* corresponding to linear DNA polymers (λ-DNA).

Scaled concentration c/c^*	Chain topology	Scaled single mode relaxation time τ_s (s cP^{-1})	Scaled fast double mode relaxation time $\tau_{d,1}$ (s cP^{-1})	Scaled slow double mode relaxation time $\tau_{d,2}$ (s cP^{-1})
10^{-5} (dilute)	circular	0.026 ± 0.005	-	-
0.025	circular	0.025 ± 0.005	0.0055 ± 0.0012	0.050 ± 0.006
0.1	circular	0.026 ± 0.006	0.0071 ± 0.002	0.049 ± 0.012
1.0	circular	0.032 ± 0.008	0.0091 ± 0.003	0.082 ± 0.016
10^{-5} (dilute)	linear	0.093 ± 0.014	-	-
0.1	linear	0.11 ± 0.01	-	-
0.5	linear	0.12 ± 0.01	-	-
1.0	linear	0.13 ± 0.02	-	-

*Data for linear polymers in semidilute unentangled solutions are taken from Hsiao et al. *Journal of Rheology* (2017).

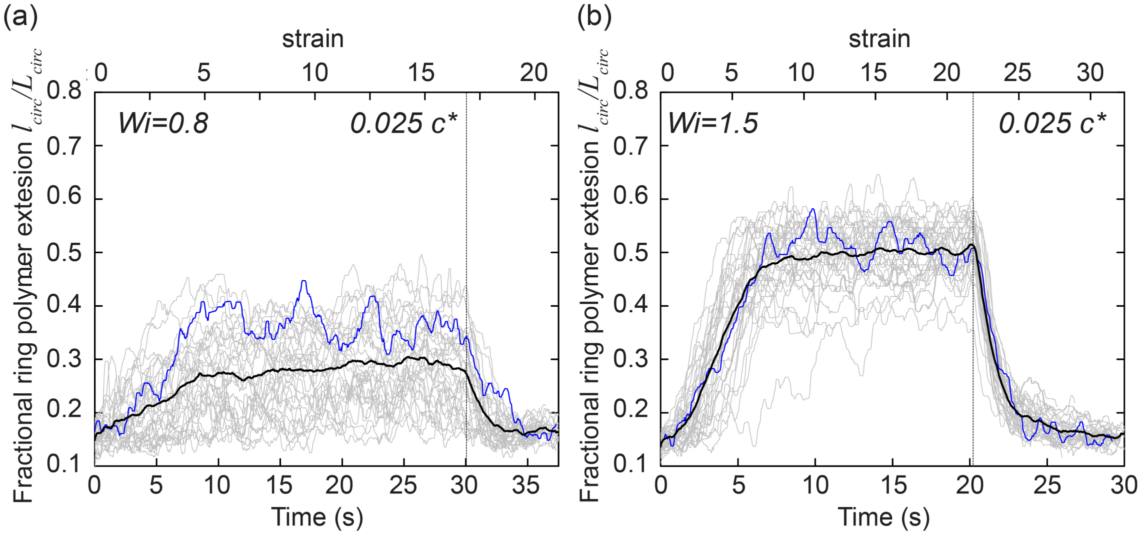

Supplementary Figure 1. Transient fractional extension of ring DNAs in semidilute unentangled linear solutions with background linear chain concentration of 0.025 c^* at (a) Wi=0.8 and (b) Wi=1.5. Molecular ensembles consist of $N = 32$ and $N = 31$ molecules respectively.

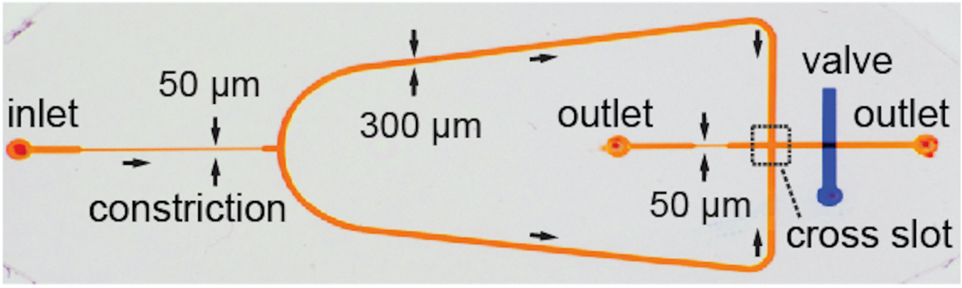

Supplementary Figure 2. Optical micrograph of the microfluidic cross-slot in which fluorescently labeled circular DNA molecules are imaged in background of semidilute unentangled DNA solutions. The microfluidic device consists of a two-layer integrated PDMS flow cell. The fluidic layer (shown in orange) has a cross-slot channel geometry to generate a planar extensional flow, and the control layer (shown in blue) contains a pressure-driven valve to control fluid flow.

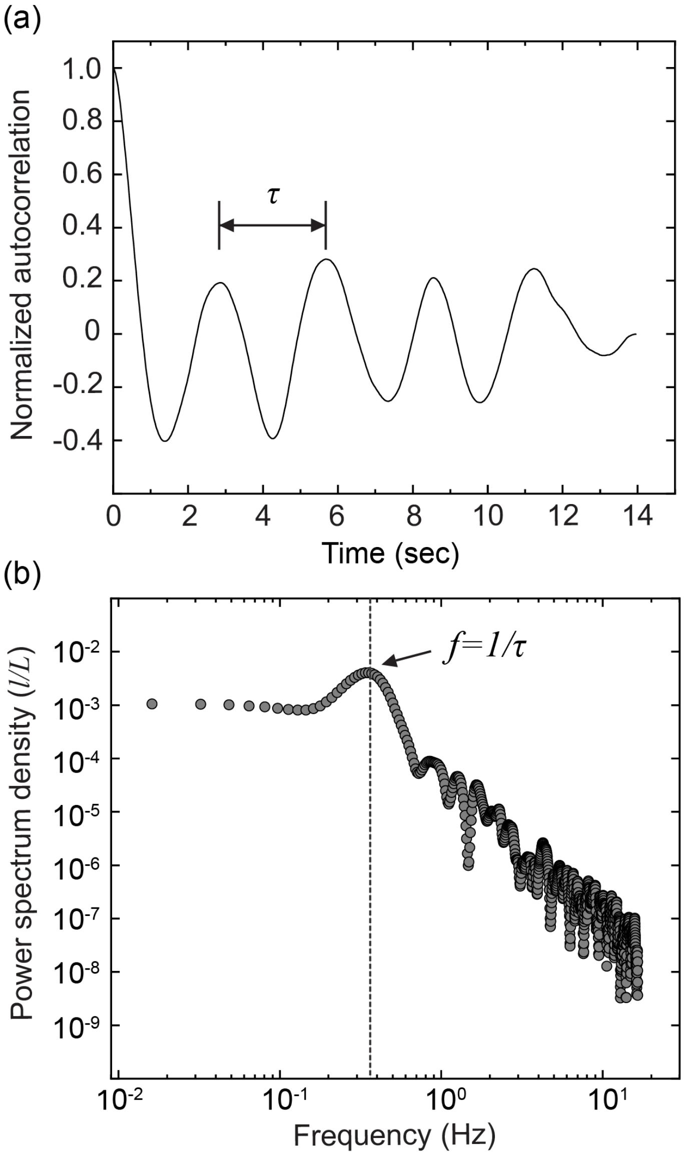

Supplementary Figure 3. (a) Autocorrelation of fluctuations in ring polymer extension after the initial transient phase for Fig. 4a at $Wi = 3.0$ and background concentration of 1.0 c^*. The characteristic timescale between peaks of the autocorrelation curve is denoted as τ, and $\tau = 2.8$ s. (b) Power spectrum density of ring polymer extension fluctuations in Fig. 4a shows a characteristic peak at frequency $f = 0.35$ Hz

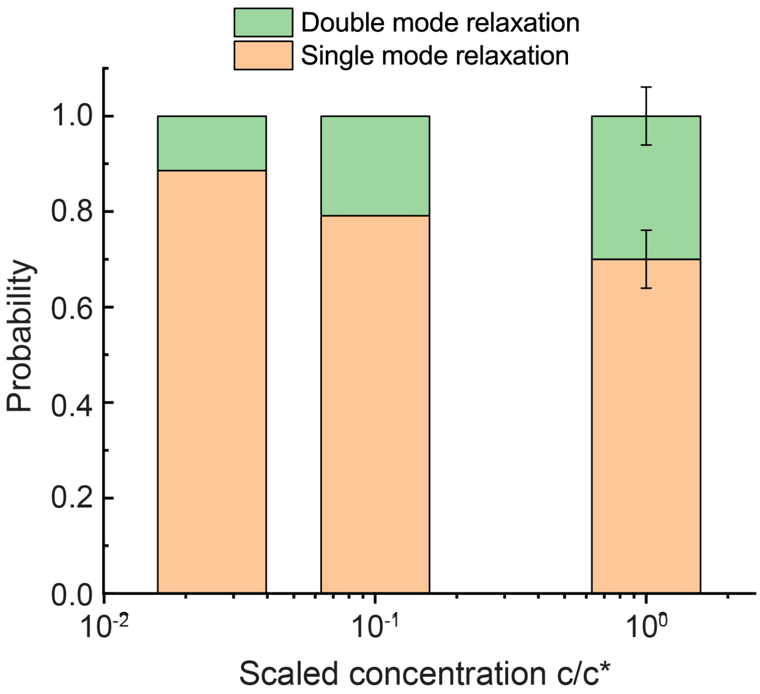

Supplementary Figure 4. Probability distribution of double mode relaxation and single mode relaxation for tracer ring polymers at $Wi = 1.5$ under different background linear polymer concentrations. Molecular ensembles consist of $N = 31$, $N = 25$, $N = 38$ molecules, respectively.

Supplementary Methods

Power spectral density (PSD). The characteristic periodic cycle of polymer motion was further characterized by the power spectral density (PSD) of projected chain extension $l(t)$. The PSD is defined as:

$$P(f) = \int_{-\infty}^{\infty} C(\lambda)_{l',l'} e^{-2i\pi f\lambda} d\lambda \tag{1}$$

where f is the frequency, λ is the offset time, $i = \sqrt{-1}$, and $C(\lambda)_{l',l'}$ is the autocorrelation function of the fluctuating quantity for the fractional extension $l'(t)$ defined in Equation 3 in the main text.
